# Sex-Related Effects of Prenatal Stress on Region-Specific Expression of Monoamine Oxidase A and β Adrenergic Receptors in Rat Hearts

**DOI:** 10.5935/abc.20190001

**Published:** 2019-01

**Authors:** Tanja Jevjdovic, Tamara Dakic, Sonja Kopanja, Iva Lakic, Predrag Vujovic, Nebojsa Jasnic, Jelena Djordjevic

**Affiliations:** 1 Faculty of Biology - University of Belgrade, Belgrado - Sérvia; 2 Department of Pediatrics and Adolescent Medicine - Medical University of Vienna, Viena - Áustria

**Keywords:** Pregnancy, Stress, Physiological, Oxidative Stress, Heart, Catecholamines, Rats, Sex, Female, Cardiotoxicity, Adrenergic beta1 beta2 Receptor Antagonists

## Abstract

**Background:**

Prenatal stress may increase risk of developing cardiovascular disorders in adulthood. The cardiotoxic effects of catecholamines are mediated via prolonged adrenergic receptor stimulation and increased oxidative stress upon their degradation by monoamine oxidase A (MAO-A).

**Objectives:**

We investigated long-term effects of prenatal stress on β (1, 2, 3) adrenergic receptors and MAO-A gene expression in the hearts of adult rat offspring.

**Methods:**

Pregnant rats were exposed to unpredictable mild stress during the third week of gestation. RNA was isolated from left ventricular apex and base of adult offspring. Quantitative PCR was used to measure gene expression in collected ventricular tissue samples. The level of significance was set to p < 0.05.

**Results:**

β3 adrenergic receptor mRNA was undetectable in rat left ventricle. β1 adrenergic receptor was the predominantly expressed subtype at the apical and basal left ventricular myocardium in the control females. Male offspring from unstressed mothers displayed higher apical cardiac β1 than β2 adrenergic receptor mRNA levels. However, β1 and β2 adrenergic receptor mRNAs were similarly expressed at the ventricular basal myocardium in males. Unlike males, prenatally stressed females exhibited decreased β1 adrenergic receptor mRNA expression at the apical myocardium. Prenatal stress did not affect cardiac MAO-A gene expression.

**Conclusions:**

Collectively, our results show that prenatal stress may have exerted region- and sex-specific β1 and β2 adrenergic receptor expression patterns within the left ventricle.

## Introduction

Emerging data from epidemiological and experimental studies have pointed out that disturbed intrauterine environment is related to the increased risk of developing pathologies later in life. Increased susceptibility to adult hypertension has been observed in offspring prenatally exposed to unbalanced maternal nutrition,^1^^-^^3^ synthetic glucocorticoids,^4^ or maternal stress.^5^ It has long been recognized that exposure to prenatal stress results in enhanced hypothalamo-pituitary-adrenal (HPA) axis and sympathetic nervous system (SNS) activity in adulthood.^6^^,^^7^

The hallmark of cardiovascular disorders is dysregulated SNS activity. Hence, it is not surprising that the key pharmaceutical targets in the management of these disorders are mostly modulators of adrenergic receptor activity. Cardiotoxic effects of catecholamines are mainly mediated via persistent or acute over-stimulation of β adrenergic receptors (ADRB).^8^ A healthy human heart expresses three ADRB subtypes, with ADRB1 being the most and ADRB3 the least abundant.^9^^,^^10^ Downregulation in the ADRB1 subpopulation is one of the molecular features of cardiac pathologies, such as human heart failure.^9^^,^^11^ Furthermore, animal transgenic studies demonstrated that early effects of ADRB2 overexpression led to increased cardiac contractility.^12^ However, later in life these transgenic animals developed ventricular dysfunction.^13^ Furthermore, another myocardial pathological condition triggered by high circulating catecholamines is defined by a region-specific, mostly apical, contractile dysfunction within the left ventricle.^14^

Additionally, cardiotoxicity may result from the production of reactive oxidative species (ROS) upon catecholamine degradation by monoamine oxidase A (MAO-A) in the heart.^15^ Cardiac MAO-A expression and activity is increased in different animal models of heart failure^16^^-^^18^ and aging.^19^

Epidemiological studies showed that female and male patients suffering from cardiovascular disease exhibit differential responsiveness to diverse recommended treatments,^20^^,^^21^ emphasizing the necessity to include both sexes in cardiovascular research.

In order to better understand molecular mechanisms by which prenatal stress may potentially contribute to the development of cardiovascular diseases in adulthood, the present study was designed to investigate region-specific gene expression of adrenergic receptor subtypes (ADRB1, ADRB2 and ADRB3) and MAO-A in the left ventricular myocardium of female and male offspring.

## Methods

### Animals

Three-month-old virgin female Wistar rats (266 ± 11.9 g) were housed with free access to food and water under constant light-dark cycle (12 h) in temperature-controlled conditions (22 ± 1°C) in the animal facility of the Faculty of Biology, University of Belgrade. Sample size was determined by convenience, and each of six pairs of female rats was caged with a sexually experienced male during a whole oestrus cycle. Day 0 of pregnancy was marked by appearance of sperm in vaginal smear. One female remained non-pregnant. To avoid selection bias, pregnant females who were mated with the same male were randomly assigned to control (n = 5) or stressed (n = 6) group and housed individually. All procedures were conducted according to the rules for animal care proposed by the Federation of European Laboratory Animal Science Associations (FELASA), and approved by the Ethics Committee of the Faculty of Biology, University of Belgrade.

### Prenatal stress protocol

During the third week of gestation (gestational day 13-20, GD13-GD20) pregnant rats were exposed to a chronic unpredictable mild stress (CUMS) protocol that included random and intermittent exposure to a variety of stressors. Detailed CUMS protocol is shown in Table 1. Briefly, animals were exposed to the following stressors in random order twice a day for 1 h or overnight: damp bedding, restraint in a Plexiglas(r) tube, cold room (4°C), cage displacement and noise, overnight illumination, and cage tilt. Control mothers were left undisturbed for the duration of their pregnancies with the exception of general handling. During the entire pregnancy, water and food intake were recorded.

**Table 1:** Stress regime

	10:00-11:00	14:00-15:00	18:00-08:00
GD14	Restraint	Damp bedding	Cage tilt
GD15	Cold room (4ºC)	Displacement and noise	Continuous illumination
GD16	Damp bedding	Restraint	Cage tilt
GD17	Displacement and noise	Cold room (4ºC)	Continuous illumination
GD18	Restraint	Damp bedding	Cage tilt
GD19	Cold room (4ºC)	Displacement and noise	Continuous illumination
GD20	Damp bedding	Restraint	Cage tilt

* GD: gestational day.

### Biochemical assays

Before first and after last exposure to the stressor, blood was collected from dam's tail vein in EDTA-containing tubes. Adrenocorticotropic hormone (ACTH) plasma levels were measured with a CLIA kit and glucose levels were measured with an Exac-tech glucose analyzer using Dextrostix reagent strips, both according to the manufacturers' instructions.

### Litters

At birth pups were counted and weighed, and litters were adjusted to eight pups with an equal number of males and females to avoid effects of litter size and litter sex-distribution on development. All pups were raised by their biological mothers. The offspring were weaned at 28 days, separated by gender and housed in groups of two per cage, according to the experimental group (C- offspring from unstressed mothers, PS- offspring from stressed mothers). Offspring's body weight and water and food consumption were recorded during both pre- and post-weaning periods. The offspring were sacrificed by decapitation at two months of age. To avoid oestrus cycle dependent fluctuations, female offspring were sacrificed in dioestrus, as confirmed by vaginal smears.

### RNA isolation

Total RNA from the basal and apical portions of the left ventricles was isolated using TRI Reagent (Sigma, Germany) according to manufacturer instructions. Total RNA concentrations were quantified by absorbance measurements at 260 and 280 nm using a spectrophotometer (Ultrospec 2000, Pharmacia Biotech, USA) according to manufacturer instructions. RNA quality was analyzed on 1.5% agarose gel containing ethidium bromide and visualized by UV transillumination (ChemiDoc-It imager, UVP, Germany).

### cDNA synthesis and quantitative real-time PCR

RNA samples (2 µg) were subjected to DNase I treatment, using rDNase I, according to manufacturer protocol (DNA-free kit, Ambion, USA). Ready-to-go You-Prime First-Strand beads transcription kit (GE Healthcare, USA) was used to generate cDNA for subsequent quantitative real-time PCR. Samples without reverse transcriptase were used to control for possible contamination of gDNA. All reactions were carried out in duplicate, using 1x TaqMan Master Mix (Applied Biosystems) and 1x TaqMan expression assays for each gene (Table 2: Adrb1, Adrb2, Adrb3, MaoA, ActB), with 2 µg of cDNA template in a total volume of 20 µl.

**Table 2:** TaqMan expression assays

Gene	TaqMan assay ID
Beta 1 adrenergic receptor (*Adrb1*)	Rn00824536_s1
Beta 2 adrenergic receptor (*Adrb2*)	Rn00560650_s1
Beta 3 adrenergic receptor (*Adrb3*)	Rn01478698_g1
Monoamine oxidase A (*Maoa*)	Rn01430955_A1
Beta-actin (*Actb*)	Rn01412977_g1

Real-time PCR reactions were performed on an Applied Biosystem 7900 Real-Time PCR System with standard PCR conditions (50°C for 2 min; 95°C for 10 min; 95°C for 15 s, and 60°C for 1 min for 40 cycles). The relative gene expression levels were determined by comparative 2ˆ(- ΔΔC_T_) quantification method^22^ using beta-actin as the reference gene.

### Statistical analysis

Statistical analysis was performed using GraphPad Prism Software-version 6.01 (San Diego, USA). Parameters measured in mothers and offspring were expressed as means ± standard deviation (SD). Data were analyzed by unpaired Student's t-test, unless otherwise indicated. Offspring data obtained by real-time PCR analysis were expressed as median with interquartile range. Two-way ANOVA analysis with Bonferroni's multiple comparison test was used to examine the effect of prenatal stress and pregnancy on maternal serum ACTH levels as well as the effects of prenatal stress on ADRB genes expression patterns in examined regions of offspring's left ventricle. The statistical significance of differences among the real-time PCR data obtained from experimental groups was evaluated by nonparametric Mann-Whitney U-test. The level of significance was set to p < 0.05.

## Results

### Effects of CUMS on maternal and offspring parameters

**Figure 1:**
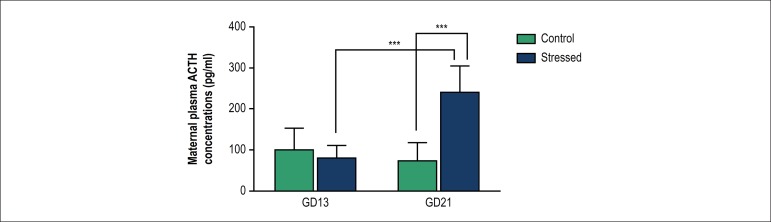
Maternal plasma ACTH concentrations before (GD13) and following (GD21) exposure to CUMS during pregnancy. Data are expressed as mean ± SD, control group (open bars, n =5), stressed group (black bars, n = 6). In the stressed group on GD21 two samples were excluded due to hemolysis. ***p < 0.001, 2-way ANOVA and Bonferroni's multiple comparison test

In order to determine whether the stress protocol applied activated HPA axis in pregnant females, maternal plasma ACTH levels were evaluated. Prior to the start of the stress protocol (GD13), maternal plasma ACTH levels were not significantly different between experimental groups (Figure 1). Following random and intermittent exposure of the pregnant female rats to a variety of stressors during the third week of gestation (GD13-21), maternal plasma ACTH levels increased compared to control pregnant females (Figure 1, p < 0.001, 2-way ANOVA with Bonferroni's multiple comparison test). Additionally, in the group of stressed mothers, following exposure to diverse stressors, plasma ACTH levels increased compared to GD13 suggesting that HPA axis was activated in this experimental group (Figure 1, p < 0.001, 2-way ANOVA with Bonferroni's multiple comparison test).

CUMS did not affect maternal weight gain during the last week of pregnancy (Table 3) or water and food intake throughout pregnancy (data not shown). Maternal blood glucose levels were similar in both experimental groups before and after application of the CUMS protocol (Table 3). There was no effect of prenatal stress on litter size or offspring sex ratio (Table 3). Maternal stress during the last week of pregnancy did not affect offspring birth weight or weight gain during either pre- or post-weaning periods (Table 4).

**Table 3:** Maternal weight before treatment, maternal weight gain during last week of pregnancy (GD13-GD21), gestation length, maternal blood glucose level before and after stress exposure, litter size, and sex ratio

Variable	Control (n = 5)	Stressed (n = 5)	p
Maternal weight before treatment (g)	347 ± 37.3	337 ± 40.2	0.6646
Maternal weight gain (g)	62.5 ± 4.43	50.5 ± 11.6	0.0872
Gestation length (days)	22.0 ± 0.71	22.2 ± 0.41	0.6355
Blood glucose levels (mM) before stress (GD13)	5.44 ± 0.21	5.55 ± 1.00	0.8162
Blood glucose levels (mM) after stress (GD21)	5.30 ± 0.42	5.63 ± 0.69	0.3737
Litter size	11.2 ± 2.77	11.8 ± 2.32	0.6891
Sex ratio	1.38 ± 0.4	1.18 ± 0.3	0.3805

GD13: gestational day 13; GD21: gestational day 21; Data are expressed as means ± standard deviation (SD).

**Table 4:** Offspring weight at birth, postnatal day 28 (PND28) and 60 (PND60)

Variable		C	PS	p
Birth weight (g)	Group	6.67 ± 0.904	6.39 ± 0.685	0.1562
Weight at PND28 (g)	Group	94.5 ± 11.4	96.9 ± 13.2	0.6360
	Male	96.8 ± 12.3	94.5 ± 10.3	0.7286
	Female	92.2 ± 11.1	99.3 ± 16.2	0.3924
Weight at PND60 (g)	Group	316 ± 50.9	317 ± 70.5	0.9790
	Male	355 ± 29.7	377 ± 42.7	0.3354
	Female	277 ± 33.6	257 ± 21.7	0.2454

C: offspring from unstressed mothers; PS: offspring from stressed mothers; PND28: postnatal day 28; PND60: postnatal day 60; Number of animals (n): n = 5-8 per group. Data are expressed as means ± standard deviation (SD).

### Effects of prenatal stress on regional ADRB subtype gene expression in left ventricle of female and male

Using quantitative PCR analysis, relative mRNA levels of ADRB1, ADRB2, and ADRB3 were examined at the apical and the basal region of left ventricle harvested from control (C) and prenatally stressed (PS) adult female and male offspring.

ADRB3 mRNA was undetectable at the examined regions of the left ventricle in male and female offspring.

**Figure 2:**
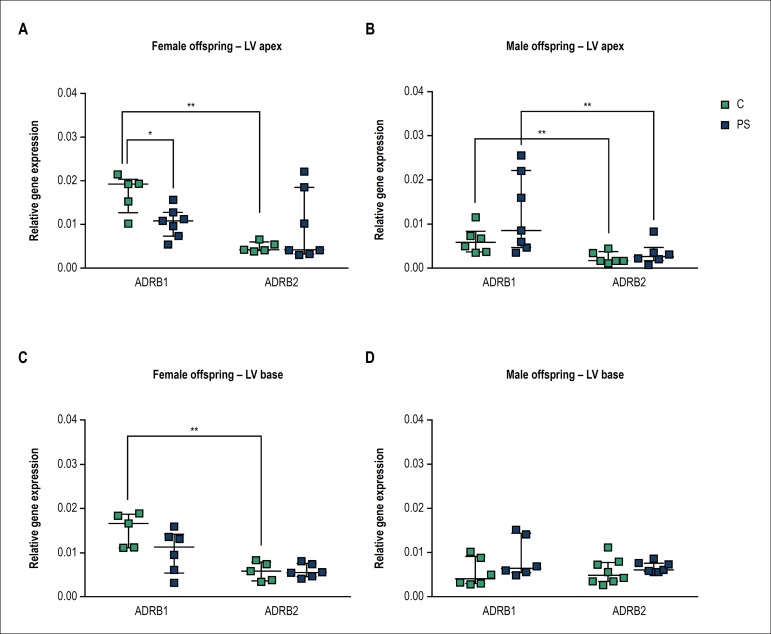
Effects of prenatal stress on expression of beta 1 (ADRB1) and beta 2 (ADRB2) adrenergic receptors mRNA at the apex and base of the left ventricle in the offspring (LV). Results are presented for female (A and C) and male (B and D) offspring from unstressed (control-C) and stressed mothers (prenatal stress-PS). Data are expressed as median with interquartile range (number of animals per group, n = 5-8 per group). *p < 0.05; **p < 0.01, Mann-Whitney U-test

We detected higher ADRB1 mRNA expression at the apex and the base of left ventricle from control female offspring, in comparison to ADRB2 mRNA levels (Figure 2A and 2C, approx. ADRB1:ADRB2 = 73%:23%, p < 0.01). Decreased apical ADRB1 mRNA levels were detected in PS females compared to control animals (Figure 2A, p = 0.048). Additionally, in PS females, we observed a trend of increase in apical ADRB2 mRNA levels compared with control. Since these changes resulted in the loss of differential ADRB subtype expression levels at the apical myocardium of PS females, two-way ANOVA analysis was performed. ANOVA test revealed significant interaction between prenatal treatment and receptor subtype expression levels (F(1,20) = 6.817, p = 0.0167). Altogether, these results indicate that prenatal stress differently affected ADRB1 and ADRB2 at the apical myocardium of female animals. Furthermore, we observed a trend of decrease in basal ADRB1 mRNA levels of PS females compared with control (Figure 2C p = 0.3434), such that basal myocardium of PS females did not display differential ADRB1 and ADRB2 mRNA expression pattern compared with control animals. One cannot exclude the effect of limited sample size to detect significant differences in ADRB gene expression between control and PS groups. Further research will be necessary to obtain a more detailed understanding of the underlying mechanisms resulting in altered gene expression pattern of basal cardiac adrenergic receptors of PS females.

Male offspring from unstressed mothers, similar to female offspring, displayed higher ADRB1 than ADRB2 mRNA levels at the apex of left ventricle (Figure 2B, p = 0.0087). However, differently from female offspring, prenatal stress did not affect the predominant apical ADRB1 mRNA expression pattern of left ventricle in male offspring (Figure 2B). On the other hand, we detected similar ADRB1 and ADRB2 mRNA expression levels at the base of the left ventricle in control and PS male offspring (Figure 2D).

### Effects of prenatal stress on regional MAO-A gene expression in left ventricle of female and male offspring

**Figure 3:**
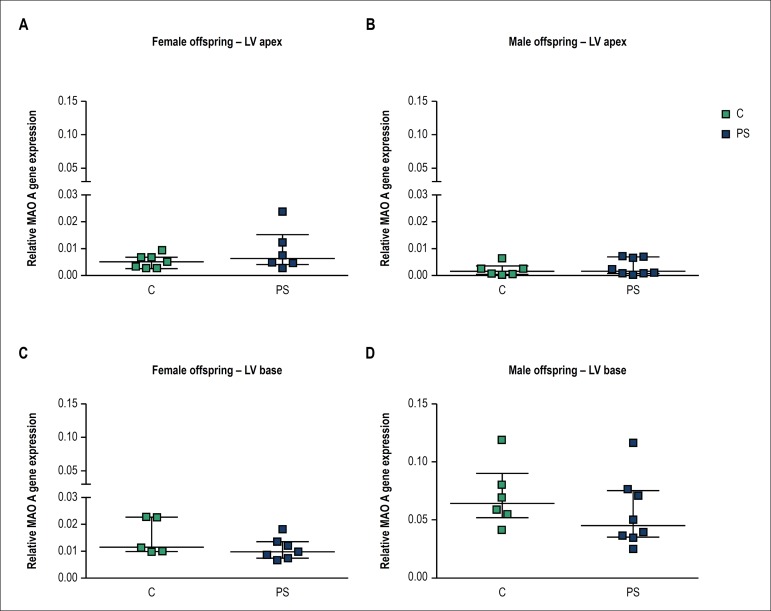
Effects of prenatal stress on monoamine oxidase A (MAO-A) mRNA at the apex and base of the left ventricle (LV) in the offspring. Results are presented for female (A and C) and male (B and D) offspring from unstressed (control-C) and stressed mothers (prenatal stress-PS). Data are expressed as median with interquartile range (number of animals per group, n = 5-8).

Prenatal stress did not significantly affect MAO-A mRNA expression at either apical or basal region of left ventricle in female and male offspring (Figure 3). Based on our results we observed a trend toward higher relative expression of MAO-A at the basal myocardium compared to the apical region of the left ventricle in male offspring (approximately 35-fold in control and 17.5-fold in PS animals, Figure 3B, D). Additionally, basal cardiac MAO-A demonstrated a trend toward higher expression in males than in females (Figure 3C, D, approximately, 4.7-fold between control groups and 5.1-fold between PS groups).

## Discussion

Cardiovascular diseases are the leading cause of morbidity and mortality worldwide.^23^ It has been shown that various disturbances of fetal development may contribute to development of cardiovascular disorders in adulthood. Offspring from stressed mothers or mothers undergoing glucocorticoid therapy during pregnancy display various neuroendocrine and behavioral alterations during adulthood.^24^^-^^26^

This study examined expression of ADRB subtypes and MAO-A in different regions of the left ventricle in the offspring of both sexes prenatally exposed to maternal stress.

We applied stress protocol to pregnant rat females that could potentially mimic everyday life stress that pregnant females are exposed to. Our stress protocol involved chronic exposure to various mild stressors which prevents habituation, which can be observed after repeated exposure to the same stressor.^27^ Plasma ACTH level was increased in stressed mothers compared to pregnant unstressed rats, which indicated that HPA axis activity of pregnant females was increased by the CUMS protocol, which is consistent with previous studies.^28^^,^^29^ We did not observe any significant difference in metabolic parameters such as maternal weight gain during pregnancy, water and food consumption, or blood glucose level between stressed and unstressed mothers. Nor did maternal stress during the last week of pregnancy affect litter size or birth weight. Taken together, these results imply that our model of CUMS was potent enough to induce a stress response in pregnant rats but did not affect offspring weight, which is known to be one of the risk factors for development of adult cardiovascular disorders.^2^

To the best of our knowledge, this is the first study to report relative gene expression levels of beta-adrenergic receptor subtypes in two different regions within rat left ventricle. Our results show that ADRB1 is the predominantly expressed subtype of the cardiac ADRB population at apical and basal myocardium of left ventricle in female rat offspring from unstressed mothers. We also detected higher expression of ADRB1 compared to ADRB2 mRNA levels at the apical ventricular region in the control male offspring. Indeed, several human and other animal studies have demonstrated higher ADRB1 than ADRB2 density in left ventricle.^30^^-^^34^ However, our results are not in accordance with the findings reported by Paur et al.,^35^ who used radioligand binding-displacement assays. They demonstrated increased ADRB2:ADRB1 ratio in the apical cardiomyocytes isolated from adult male Sprague Dawley rats. This discrepancy may be accounted for by different methods and model systems. Differently from in female offspring, ADRB1 and ADRB2 mRNA levels were similarly expressed in the left ventricular basal myocardium in male rat offspring. We did not detect ADRB3 mRNA in rat left ventricle.

Our results suggest that there are sex- and region-specific gene expression representations of ADRB subpopulations within left ventricular rat myocardium. Additionally, data from our study indicate that prenatal stress may have affected ARB1 and ARB2 gene expression pattern at the apical region of the left ventricle in female offspring, but not in male offspring. Disturbed representation of cardiac adrenergic receptors subtypes has been described in cardiovascular pathologies. Heart failure is characterized by altered ADRB1:ADRB2 ratio, in part due to the decreased ADRB1 protein and mRNA within left ventricle.^11^^,^^36^ The nonselective reduction of beta-adrenergic receptor subpopulations was also observed in the heart of both aged animals^37^ and elderly patients.^31^^,^^34^ Our results indicate that prenatal stress resulted in decreased apical ADRB1 mRNA expression suggesting that apical myocardial region of the female rat offspring might be sensitive to stress exposure during fetal life. Interestingly, higher sensitivity of the apical region within left ventricle to stress during adulthood has been described in Takotsubo (stress-induced) cardiomyopathy.^35^^,^^38^ Moreover, this syndrome is predominantly diagnosed in women.^38^

Another protein that is involved in the sympathetic modulation of cardiac function is MAO-A. This enzyme catalyses the oxidation of monoamines during which ROS is produced and may contribute to the pathogenesis of cardiovascular diseases.^15^ To the best of our knowledge this is the first study to investigate the effects of prenatal stress on cardiac MAO-A gene expression in the offspring. In the present study we did not detect significant changes in the MAO-A mRNA levels in the prenatally stressed heart of either sex.

There are several limitations to this study. As mentioned above we cannot exclude the effect of limited sample size on detecting additional significant differences in region specific gene expression of myocardial beta-adrenergic receptor subpopulations. The mechanism for decreased apical myocardial ADRB1 mRNA expression in prenatally stressed female, but not male, offspring is unknown. We can only hypothesize based on available literature that sex hormones might have an effect. Thus, it would be of interest to investigate earlier developmental stages of prenatally stressed offspring. Furthermore, we did not compare cardiac expression levels of MAO-A between male and female offspring. However, based on the relative expression levels of MAO-A, we can hypothesize that our results suggest that cardiac MAO-A exhibits a sex dimorphic gene expression pattern, which is likely expressed more abundantly in the heart of male rats than in female rats. As MAO-A is a main source of hydrogen peroxide in the heart, our observation would be in agreement with the reported lower production of hydrogen peroxide in cardiac mitochondria of female, compared to male Wistar rats.^39^

## Conclusions

In summary, our data suggest that prenatal stress may exert, already at young adult age, sex-specific changes in apical and basal cardiac adrenergic receptor subpopulations in offspring. Whether these changes correlate with diminished cardiac performance and predispose organisms to develop cardiovascular diseases during their lifetime remains to be determined in future experiments.
